# Stereotypical force patterns of the elephant trunk in planar reaching movements

**DOI:** 10.1016/j.isci.2026.115108

**Published:** 2026-02-23

**Authors:** Camilla Agabiti, Enrico Donato, Elisa Setti, Paule Dagenais, Michel C. Milinkovitch, Cecilia Laschi, Angelo Maria Sabatini, Barbara Mazzolai, Egidio Falotico

**Affiliations:** 1 BRAin-Inspired Robotics (BRAIR) Lab, The BioRobotics Institute, Department of Excellence in Robotics and AI, Sant’Anna School of Advanced Studies, 56025 Pontedera, Italy; 2 Physics of Cells and Cancer, Institut Curie, Université Paris Sciences et Lettres (PSL), 75005 Paris, France; 3 Laboratory of Artificial and Natural Evolution (LANE), Department of Genetics and Evolution, University of Geneva, Swiss Institute of Bioinformatics (SIB), 1211 Geneva, Switzerland; 4 Department of Mechanical Engineering and Advanced Robotics Centre, National University of Singapore, Singapore 119077, Singapore; 5 Bioinspired Soft Robotics Laboratory, Istituto Italiano di Tecnologia, 16163 Genoa, Italy

**Keywords:** applied sciences, engineering, robotics

## Abstract

The elephant trunk is a highly dexterous muscular hydrostat whose continuous, distributed deformations pose significant challenges for mathematical modeling. We introduce linear “stereotypical” laws that map desired trunk configurations, parameterized by curvature and length, directly to the internal muscle-analogue forces required in our rod-based dynamic model. The trunk is represented as a simplified multi-segment structure of point masses linked through longitudinal and radial muscle analogues and connective tissue, all modeled using rods. Using these laws, the model predicts biological reaching trajectories with tip-position errors below 8% while maintaining hydrostatic volume across trials. The resulting force-shape mappings reveal consistent, repeatable internal force patterns underlying trunk postures, providing a compact representation of actuation strategies that generate specific planar shapes. By reducing high-dimensional continuum dynamics to simple linear relationships, this framework preliminarily enables the inference of muscle-force distributions from shape configurations, laying the groundwork for deeper exploration of the elephant trunk motion strategies and their translation into advanced robotic systems control.

## Highlights

• A rod-based dynamic model is developed to study planar reaching in the elephant trunk
• Linear laws map trunk curvature and length to internal muscle-analogue forces
• Force correlations reflect muscular principles underlying elongation and bending

## Graphical abstract

**Figure:**
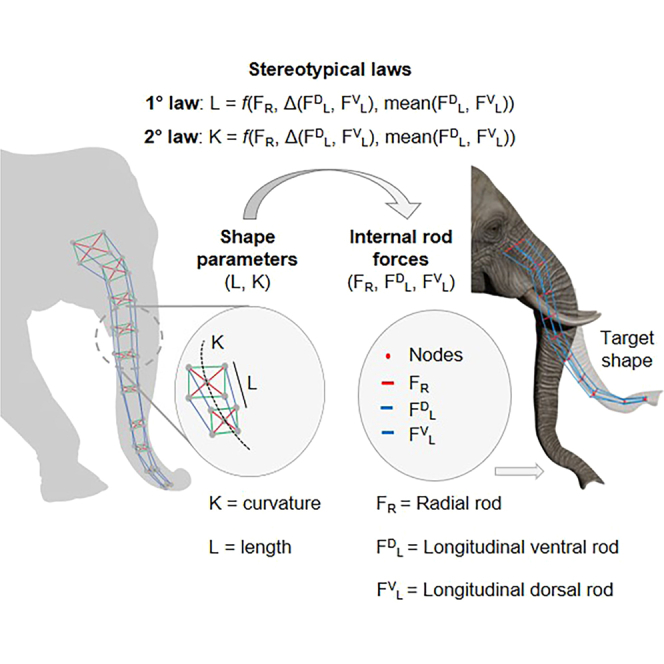

## Introduction

The elephant trunk is a continuous, skeleton-free biological structure that relies on muscles for both support and movement. Systems exhibiting this morphology are referred to as *muscular hydrostats*.^1^ Muscular hydrostats are characterized by closely packed three-dimensional arrays of muscle bundles which are oriented in three main directions: i) longitudinal muscles, which are parallel to the long axis, ii) radial (and transverse) muscles, perpendicular to the long axis, and iii) oblique muscles, helical around the long axis. All possible motions are generated by combinations of four elementary motions, which are elongation, shortening, bending and torsion.^2^ By combining these, the trunk achieves exceptional dexterity, making it one of the most versatile hydrostats in nature.^3^ Continuous structures, unlike rigid bodies constrained by a finite number of degrees of freedom (DoFs), exhibit distributed deformations, enabling them adaptability in complex environments.^4^^,^^5^ This unique characteristic has fueled advancements in soft and bioinspired robotics, such as robotic analogs of the seahorse tail,^6^ elephant trunk,^7^ or octopus arm.^8^^,^^9^ Continuous biological systems have been modeled to uncover their biomechanics or to extract principles for engineering applications.^10^ To emulate these biological control principles, recent works have used Cosserat rod theory and continuum mechanics approaches. Notable examples include dynamic models of octopus arms with distributed muscle-like actuation,^11^^,^^12^^,^^13^^,^^14^^,^^15^ and 2D or 3D biomimetic arm models for simulating hydrodynamics,^16^^,^^17^^,^^18^ as well as energy-efficient task-specific movements.^19^^,^^20^ Similar frameworks have been applied to the modeling of annelids,^21^ leeches,^22^ reptilian tongues,^23^ and squid tentacles,^24^ often aiming to simulate neuromuscular coordination and task-level biomechanics.^25^

While these studies offer insight into the biomechanics of flexible systems, a major open question remains: how to derive generalizable mappings between shape configurations and internal actuation forces in such continuum structures. Tekinalp et al.^26^ present a study on the topology, dynamics, and control of a muscle-architected soft robotic arm inspired by muscular hydrostats. In particular, they analyze how the internal arrangement affects the system’s behavior, and propose a control framework to achieve complex motions in grasping and manipulation tasks. Recent work has also deepened our understanding of the elephant trunk’s structure and biomechanics from both experimental and computational perspectives. Experimental studies,^3^^,^^27^^,^^28^^,^^29^ and modeling works^30^^,^^31^^,^^32^^,^^33^ provided foundational knowledge of its morphological properties, qualitative motion principles, as well as muscles synergies underlying motion generation. Recently, Kaczmarski et al.^34^ developed a reduced-order model of the elephant trunk, introducing a minimal mechanical formulation to capture its fundamental movement capabilities. Their approach identifies the minimal set of mechanical principles necessary to describe trunk motions in quasi-static tasks. Despite these advancements, accurately capturing the dynamics of the elephant trunk remains a challenge. Moreover, these models lack the ability to generalize force coordination principles across movement types or to provide interpretable mappings between motion and actuation.

In this work, we aim to address this gap by focusing not only on trunk simulation but rather on the extraction of stereotypical linear laws that map desired trunk configurations, expressed in terms of curvature and length, to the internal forces responsible for generating them. To achieve this, we develop a 3D dynamic trunk model based on rod theory, which serves as a computational tool for replicating reaching behaviors and retrieving force patterns across trunk segments. The resulting mappings provide biologically grounded approximations of muscle coordination and offer a reduced-complexity framework for force prediction. These contributions represent a step toward linking motion generation and actuation in the elephant trunk, with direct relevance to model-based control strategies for soft robotic systems.

## Results

With our results, we demonstrate that the proposed model is able to capture the complex dynamics of the elephant trunk during planar reaching movements. The simulated trajectories replicate biological observations, reproducing both pure bending (B) and combined bending-elongation (BE) deformations. Beyond replicating kinematics, model selection analyses have revealed that the dynamic model performance improves when considering a decreasing Young’s modulus from base to tip. Furthermore, the model also preserves volume across trials and with respect to biological reference, aligning with the constant-volume characteristic of muscular hydrostats. Aiming to understand the relationships among all the involved variables (trunk shape parameters and internal rod forces), we performed a multilinear correlation analysis; we observed a strong inverse correlation between radial and longitudinal forces, a positive correlation between segment length and radial force, and a clear link between curvature and differential longitudinal forces. These insights converged into a set of predictive, low-dimensional equations, named as *stereotypical laws*, that map shape parameters to internal rod forces. When deployed in simulation, these laws compute the internal rod forces required to generate desired trunk configurations.

### Comparison between simulation results and experimental data

**Figure 1:**
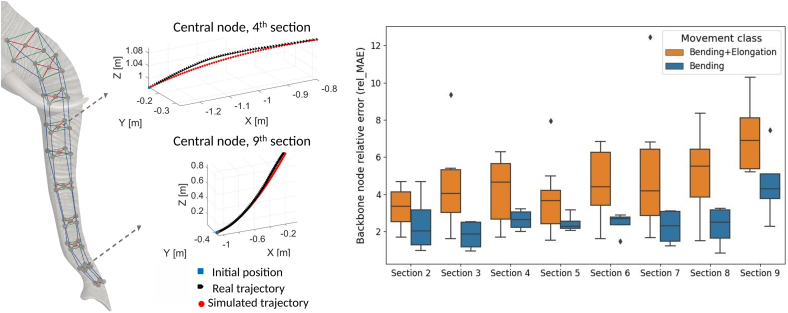
Comparison between simulation results and experimental data On the left: simulated vs. real trajectories of the backbone nodes of trunk sections 4 and 9. On the right: box-plot shows the backbone node relative errors of each trunk section for the two movement classes. Boxes indicate median and interquartile range; whiskers indicate minimum and maximum values.

To assess the predictive accuracy of the 3D dynamic model, we analyzed two classes of reaching movements: bending combined with elongation (BE) and pure bending (B). Figure 1 presents an overview of the model performances. The left panel shows a qualitative comparison between simulated and real trajectories for the central node of trunk sections 4 and 9 for a B movement. As it is visible from the plots, the simulated trajectories are qualitatively similar to the real ones.

To provide a quantitative assessment of trajectory accuracy, we computed the relative mean absolute error (rel_MAE) for each trunk section: the relative mean absolute error is computed as the mean absolute error of the node divided by the length of the corresponding node trajectory. The boxplot on the right part of Figure 1 compares the trajectory errors for BE and B movements across all trunk sections. The results highlight that trajectory errors are low in the proximal regions but progressively increase along the trunk length, reaching their peak at the tip. This trend is consistent across both movement classes, with movements generally exhibiting higher variability compared to B movements. To further quantify these errors, Table 1 summarizes the average trajectory deviations at the trunk tip across multiple trials. The results show that BE movements yield an MAE of 0.0441 m and an average rel_MAE of 5.23%, while B movements exhibit an MAE of 0.0365 m m and an average rel_MAE of 4.52%. The BE movements exhibit slightly higher relative errors, suggesting that the inclusion of elongation may contribute to decreasing model performances. Although deviations in the order of 4 cm may appear significant, they remain within the physical radius of the trunk tip itself (around 5 cm). Necessarily, this error is also influenced by the cumulative effect of small discrepancies propagating along the multisegment structure from base to tip. Despite the inherent complexity of the model, the predictions remain reasonably accurate, with error levels below 5.5% in both movement categories.

**Table 1:** Average values of mean absolute errors (MAEs) and relative mean absolute errors (RMAEs) for BE and B reaching movements

Movement Class	Trials	Avg. MAE_tip (m)	Avg. RMAE_tip (%)
BE	8	0.0411	5.23
B	4	0.0365	4.52

### Model selection results

To reflect the natural stiffness gradient of the elephant trunk, which is stiffer proximally and more compliant distally, we performed a sensitivity analysis by varying the Young’s modulus along the trunk. We conducted new simulations for all reaching movements using updated Young’s modulus values (see Table S1 in the Appendix), and we evaluated the corresponding errors at the central node of each of the nine trunk segments. Simulations showed that a decreasing stiffness profile improved trajectory predictions, particularly in proximal segments, with Case 3 yielding the most significant error reductions (up to 14.8%). In contrast, applying minimal stiffness distally (Case 2) slightly worsened performance in the last segment. Overall, incorporating a longitudinal stiffness gradient enhanced model accuracy compared to a constant stiffness configuration.

### Constant volume preservation

**Figure 2:**
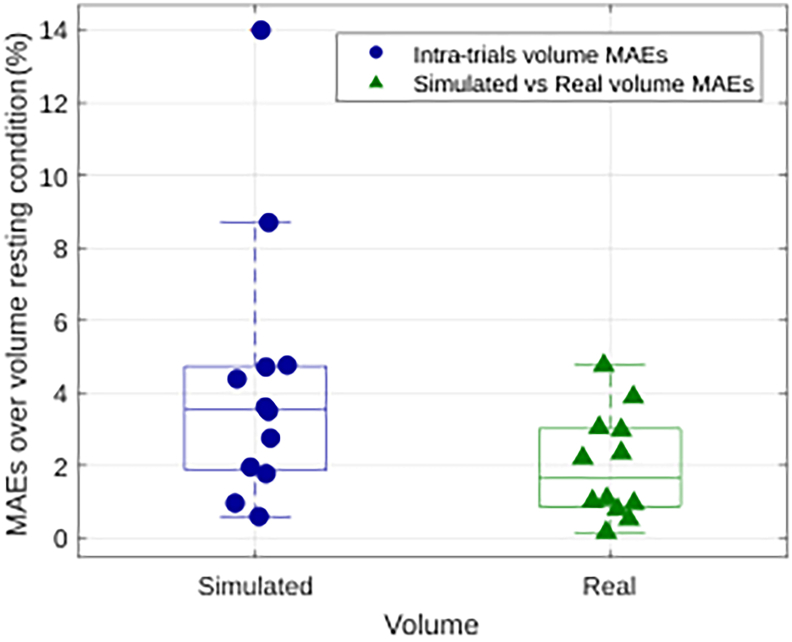
Constant volume preservation Boxplot shows trunk volume variability in simulated trials and with respect to real trunk volume. Boxes indicate median and interquartile range; whiskers indicate minimum and maximum values.

The elephant trunk functions as a muscular hydrostat, maintaining a nearly constant volume during motion. This biomechanical constraint implies that any local compression along one axis must be balanced by an expansion along another, resulting in a coordinated interplay between radial and longitudinal muscle contractions. Constant volume preservation of the trunk is an additional result of our model. As shown in Figure 2, the variability of both simulated and real trunk volumes is plotted across multiple trials, with the results expressed as the percentage mean absolute error (MAE) relative to a reference resting trunk volume.

**Figure 3:**
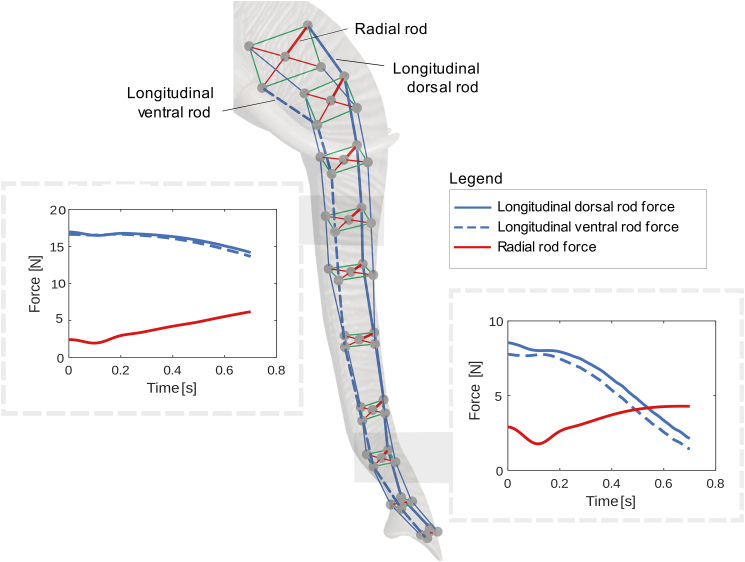
Constant volume preservation Temporal evolution of forces in the radial and longitudinal rods for two trunk sections.

The data indicate that the simulated volumes closely follow the variability pattern of the real volumes. Both distributions show minimal trial-to-trial deviations, highlighting the model’s robustness in maintaining constant trunk volume during motion. This consistency demonstrates the model’s capability to replicate realistic dynamic movements of the trunk. Additionally, the trunk’s constant volume is preserved through the reciprocal interaction between the radial and longitudinal rods of the trunk segments. Figure 3 presents the temporal evolution of forces in the radial and longitudinal rods for two different trunk segments, one proximal (top) and one distal (bottom). The forces exerted by the longitudinal dorsal and ventral rods, as well as the radial rod, exhibit an inverse relationship over time. Specifically, as the force in the radial rod increases, the forces in the longitudinal rods decrease, and vice versa. This antagonistic relationship between the forces in the radial and longitudinal rods is consistently observed across both the proximal and distal segments of the trunk. This interplay of forces is essential for maintaining the constant-volume constraint during planar reaching movements.

### Evaluation of multilinear correlation models for force-shape relationships

To further investigate the relationship between internal forces and trunk morphology, we conducted a pairwise linear correlation analysis among the main variables considered in this study: segment curvature (K), segment length (L), radial rod force $\left(F_{R}\right)$, the difference between dorsal and ventral longitudinal forces $\left(\Delta F_{L}\right)$, and the mean longitudinal force $\left(F_{m\text{\_}L}\right)$. In our modeling framework, these internal forces are the output of the dynamic simulation. The objective of this initial analysis was to explore the linear correlations between all combinations of variables, providing an overview of the underlying relationships in the system. This step served as a basis for assessing the existence of stereotypical patterns, thus motivating the derivation of simplified laws linking shape parameters to internal forces. The results are shown in Figures S7–S10 in the Appendix. Specifically, Figures S7 and S8 present the correlation matrices for a proximal and a distal segment, respectively, during pure B movements; the results for combined BE movements are shown in Figures S9 and S10. The analysis revealed a strong positive correlation between radial force and segment length, consistent with the hypothesis that radial contraction contributes to axial elongation. Conversely, a strong negative correlation between radial and mean longitudinal forces was observed, confirming the expected antagonistic behavior characteristic of muscular hydrostats. In addition, the curvature of the segments showed a positive correlation with $\Delta F_{L}$, indicating that differential longitudinal activation plays a primary role in bending dynamics. Notably, in BE movements, the correlation between radial force and segment length was even more pronounced in both proximal and distal segments, suggesting enhanced muscle coordination during combined deformations. Building on these findings, we investigated whether segment curvature and length could be predicted from the forces exerted by the muscle-like rods. This analysis enabled the definition of simplified mathematical models that describe how specific internal force patterns shape the trunk’s configuration. The results of the multilinear regression analysis for curvature K and length L across different trunk segments reveal varying degrees of model performance, as reflected in the RMSE values.

#### Curvature

**Figure 4:**
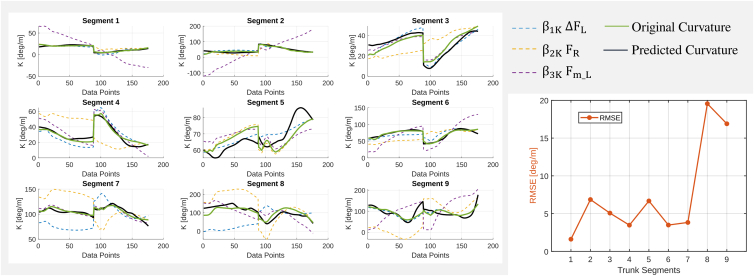
Multilinear regression model for each segment curvature Left: Comparison of predictors with curvature for each trunk segment. Right: RMSE values indicate model performance.

The multilinear regression model related to the factors influencing segment Curvature (K) involves the force difference between the longitudinal dorsal and ventral rods $\left(\Delta \;F_{L}\right)$, the radial rod force $\left(F_{R}\right)$, and the mean longitudinal force $\left(F_{m\text{\_}L}\right)$ as predictors. The results of the regression analysis indicate that the model’s performance varies across different trunk segments. As illustrated in Figure 4, the model captures curvature trends in most segments, with the predicted curvature (black line) following the original curvature (green line).

This confirms a strong relationship between K and the selected predictors: the force difference between the longitudinal dorsal and ventral rods $\left(\Delta \;F_{L}\right)$, the radial rod force $\left(F_{R}\right)$, and the mean longitudinal force $\left(F_{m\text{\_}L}\right)$. However, model accuracy is not uniform across all segments. In some cases, discrepancies between predicted and observed curvature are more pronounced, suggesting that the model may not fully capture the underlying biomechanical interactions. The root-mean-square error (RMSE) analysis further supports these observations, highlighting variability in prediction error across segments. Notably, segments 8 and 9 show significantly higher RMSE values, suggesting greater uncertainty in curvature predictions for these regions. These discrepancies may be attributed to nonlinear force interactions or additional unmodeled factors influencing segment deformation. Furthermore, greater variability in the distal segments is expected, as curvature errors accumulate along the trunk, amplifying deviations closer to the tip.

#### Length

**Figure 5:**
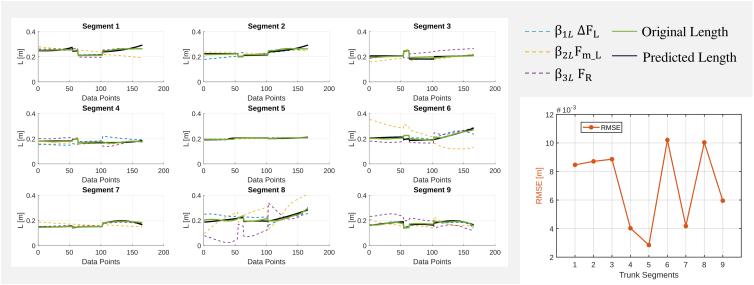
Multilinear regression model for each segment length Left: Comparison of predictors with length for each trunk segment. Right: RMSE values indicate model performance.

The multilinear regression model analyzing the factors influencing segment length (L) includes the force difference between the longitudinal dorsal and ventral rods $\left(\Delta \;F_{L}\right)$, the radial rod force $\left(F_{R}\right)$, and the mean longitudinal force $\left(F_{m\text{\_}L}\right)$ as predictors. The model performance varies across trunk segments, as illustrated in Figure 5. In most segments, the predicted length (black line) follows the original length (green line), confirming that the selected predictors are sufficient to capture the primary factors determining segment elongation. However, while some segments exhibit a strong agreement between predicted and observed length, others show deviations, suggesting that additional mechanical factors or nonlinear effects may be influencing segment deformation. RMSE analysis further supports these findings, highlighting variability in prediction accuracy across segments. In most cases, RMSE remains relatively low, reinforcing the robustness of the force-length relationship. However, certain segments, particularly 6 and 8, exhibit high RMSE values, indicating greater uncertainty in length predictions. As with curvature, it is expected that prediction variability increases toward the tip of the trunk due to the accumulation of small errors along the structure. This suggests that additional unmodeled biomechanical factors may play a role in determining segment length, particularly in distal regions.

#### Radial force

**Figure 6:**
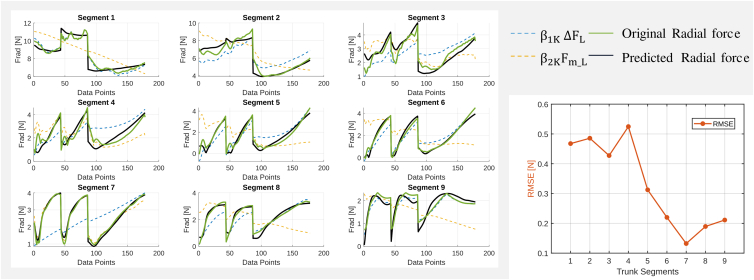
Multilinear regression model for each segment radial force Left: Comparison of predictors with radial force for each trunk segment. Right: RMSE values indicate model performance.

The final multilinear regression analysis focuses on predicting radial rod force $\left(F_{R}\right)$ using the force difference between the longitudinal dorsal and ventral rods $\left(\Delta F_{L}\right)$ and the mean longitudinal force $\left(F_{m\text{\_}L}\right)$ as explanatory variables. As illustrated in Figure 6, the predicted radial force shows a similar trend to the original radial force (green line) across most segments. However, while the model generally performs well, some deviations between predicted and observed values are evident, particularly in segments where force variations are more pronounced (e.g., segments 1, 2, 3, and 4). The RMSE analysis further supports these findings, showing that prediction errors remain relatively low across most trunk segments. Notably, RMSE values are higher in the proximal segments (e.g., segment 4) and decrease in the distal segments, indicating more reliable predictions toward the trunk tip.

### From trunk shape parameters to stereotypical force patterns

**Figure 7:**
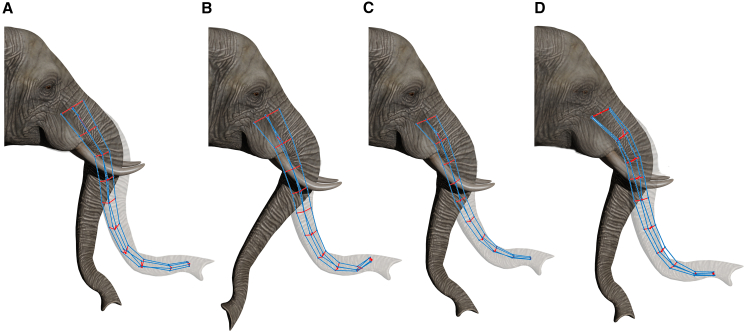
Comparison between initial, desired, and simulated trunk configurations for four B trials Panels A–D represent four different bending trajectories of the elephant trunk. The initial and final configurations of the real elephant trunk are reconstructed from experimental backbone data and visualized using Blender, with the final configuration represented in semi-transparency. The simulated trunk is shown in the final configuration reached through the use of the stereotypical laws.

**Figure 8:**
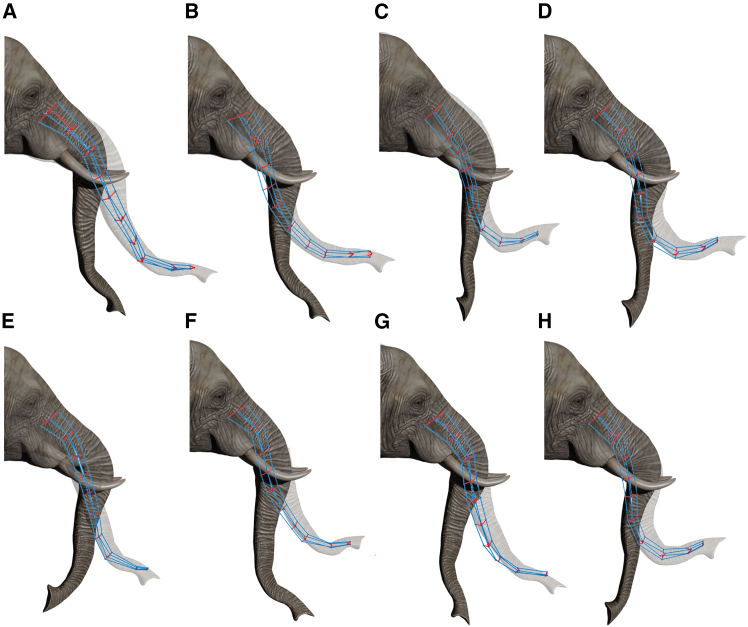
Comparison between initial, desired, and simulated trunk configurations for eight BE trials Panels A–H represent eight different bending trajectories of the elephant trunk. The initial and final configurations of the real elephant trunk are reconstructed from experimental backbone data and visualized using Blender, with the final configuration represented in semi-transparency. The simulated trunk is shown in the final configuration reached through the use of the stereotypical laws.

In this section, we present the results obtained using our approach, which consists of applying stereotypical laws derived from multilinear correlation models to estimate the internal rod forces required to reproduce specific trunk configurations in B and BE movements. Figures 7 and 8 illustrate the simulation results for these movement classes, comparing the initial shape, the desired final shape, and the final shape obtained from the simulation by applying the stereotypical laws.

Figure 7 presents four sample trials of B movements, where the trunk deforms without significant elongation. In simulations, the internal force patterns required to replicate the specific trunk configurations were computed by applying the stereotypical linear laws for segment curvature, length, and radial force, whose coefficients were derived from the multilinear correlation analysis (see Materials and Methods chapter). The computed forces are then provided as inputs to the dynamic model (see Tables S2–S4 in the Appendix). The results indicate that the model captures the expected curvature evolution, with the simulated trunk following the desired final shape across different trials.

The smooth trajectory transitions suggest that the estimated internal forces can replicate the expected muscle activation patterns. However, a qualitative comparison reveals the presence of discrepancies primarily in the distal segments of the trunk. These deviations suggest that the regression model describes the force-to-curvature relationship but may be sensitive to variations in the distal flexibility of the trunk. Overall, the model performs well in predicting bending dynamics, supporting the validity of the stereotypical laws used for force estimation. Figure 8 presents eight sample trials where the trunk undergoes BE movements. Compared to pure B movements, these trials exhibit a more complex deformation pattern. The results indicate that the model predicts both bending and length changes, maintaining smooth shape evolution throughout the reaching motion. However, discrepancies between the simulated and desired shapes are more pronounced in BE movements than in pure B cases, particularly in distal segments undergoing large deformations.

### Model performance: Error metrics

To quantitatively assess these deviations, Table 2 reports the Mean Absolute Error (MAE) for curvature and segment length across different trunk segments. The curvature MAE increases along the trunk length, starting from 2.14 *deg/m* in segment 1 and reaching 8.89 *deg/m* in segment 9 for bending movements, and from 2.018 *deg/m* to 15.6 *deg/m* in BE movements. The higher curvature errors in the distal segments suggest that elongation dynamics introduce additional nonlinearities that the regression model does not fully capture. Additionally, inaccuracies in force predictions may accumulate along the trunk, amplifying curvature deviations over time and going from the proximal part toward the distal end.

**Table 2:** Average MAE values for B and BE movements and for each trunk segment. Relative MAEs are MAEs related to the segment length

Metric	Seg. 1	Seg. 2	Seg. 3	Seg. 4	Seg. 5	Seg. 6	Seg. 7	Seg. 8	Seg. 9
MAE K (B)	5.72	8.59	0.57	13.7	15.5	34.3	37.6	36.2	39.5
rel MAE K (B)	2.14	3.71	3.11	3.74	4.10	6.86	7.52	7.24	8.89
MAE K (BE)	10.9	8.02	10.3	13.2	31.5	39.7	46.1	55.5	58.1
rel MAE K (BE)	2.018	3.60	4.03	5.64	8.80	8.94	9.22	11.1	15.6
MAE L (BE)	6.65e-3	6.89e-3	6.38e-3	7.78e-3	5.38e-3	10.8e-3	10.9e-3	18.8e-3	20.8e-3

K is measured in *deg/m* and L is measured in *m*.

In contrast, the length MAE remains relatively low, with values on the order of $10^{-3}$ *m*. The highest length error is 0.0220 *m* in segment 9, but most other segments exhibit errors below 0.010 *m*, indicating a sufficiently accurate elongation prediction. Given that the segment length is approximately 0.2 *m*, these errors are considered acceptable within the context of our model.

To quantify discrepancies between simulated and experimental trajectories, we computed the Mean Absolute Error (MAE) and the Root Mean Squared Error (RMSE) across all trunk nodes in both the B and BE tasks. The MAE is reported in its 2D Euclidean form, accounting for both X and Z directions. Full numerical results are provided in Tables S5 and S6 (Appendix). A consistent pattern emerges across all nodes: both MAE and RMSE increase from proximal to distal segments, indicating a gradual decrease in model accuracy toward the tip. This behavior is expected, as cumulative uncertainties, modeling simplifications, and approximations during data processing tend to amplify in the more compliant distal region. In all cases, RMSE values exceed MAE, as expected from their definitions, and the systematic proximity between the two metrics indicates that errors are relatively homogeneous across repetitions rather than dominated by isolated outliers. This suggests a systematic, model-level limitation in capturing distal kinematics. This interpretation is supported by the behavior of the adjusted coefficient of determination $\left(R_{\text{adj}}^{2}\right)$ obtained from the curvature regression model. As shown in Figure S11 in the Appendix, $R_{\text{adj}}^{2}$ values remain high in proximal segments (1–4), but drop markedly in more distal segments (7–9), confirming that the current predictors become progressively less informative, and aligning with the observed increase in positional errors.

### Limitations of the study

First, while the predicted curvatures provide a reliable approximation of the desired trajectories, discrepancies still arise. These are partly due to the use of simplified linear correlation laws, which may not fully capture the nonlinearities of muscle-force interactions. Additional sources of error include uncertainties in stiffness parameter estimation, the geometric approximations involved in the ellipse-fitting of marker sections, and the assumption of strictly planar movements, which do not strictly align with the conditions of the experimental trials. These simplifications were necessary trade-offs to ensure computational feasibility and to keep the model analytically tractable. The discrete modular representation of the trunk, as opposed to a fully continuous model, was adopted to balance these competing demands.

The combined analysis of MAE, RMSE, and $R_{\text{adj}}^{2}$ reveals systematic discrepancies between simulated and real trajectories, particularly in the distal region. The close MAE-RMSE values across all nodes indicate errors of comparable magnitude rather than outlier-driven deviations, while the decrease in $R_{\text{adj}}^{2}$ shows that the model captures trunk dynamics more accurately in proximal segments. However, this distal degradation is consistent with known mechanical constraints and does not undermine the overall ability of the model to capture the main features of proximal trunk dynamics.

A further limitation concerns the nature of the ground-truth data used to evaluate model accuracy. Markers in the dataset were placed exclusively on the dorsal side of the trunk because the animals’ tactile sensitivity prevented reliable ventral tracking. As a result, the 3D geometry of the trunk was reconstructed by fitting ellipses to each row of dorsal markers, under the assumption of elliptical cross-sections. This fitting procedure provided estimates of the ventral side and the trunk backbone.

Furthermore, at present, there are no published benchmarks or datasets reporting trajectory-level errors in real elephant trunk movements with which to directly compare our results. This lack of reference data makes it challenging to contextualize the model’s accuracy within the broader field of biological modeling.

The distal accuracy of the model represents an additional limitation. The observed average tip position error may be acceptable for planar reaching tasks, where the goal is to reproduce the overall trajectory of the trunk, but it would be insufficient in contexts requiring fine spatial precision, such as grasping.

These observations point to possible directions for refinement, including incorporating more complex force distributions, adaptive stiffness parameters, or improved segment-level control strategies.

It is important to clarify that the model does not attempt to replicate the full anatomical and mechanical complexity of the elephant trunk. Instead, it captures the main mechanical effects of muscle actions, bending, and elongation, rather than modeling every anatomical muscle group. While this abstraction reduces biological fidelity, it offers an interpretable and computationally efficient tool, well-suited for potential applications in predictive modeling and soft robot control.

## Discussion

The presented study focused on replicating planar reaching movements of the elephant trunk, targeting the macroscopic deformations that characterize its natural motions, namely bending and combined bending-elongation. By integrating a dynamic simulation approach, we were able to reproduce these reaching behaviors with acceptable accuracy, as demonstrated by the low relative trajectory errors across multiple trials. Moreover, the model successfully preserved the trunk’s constant volume during motion, reflecting the characteristic constraint of muscular hydrostats as a result of the antagonistic interplay between radial and longitudinal muscle forces, which exhibit an inverse relationship. This reinforces the biological plausibility of the simulated behavior. A fundamental advantage of our model is its dynamic formulation. Unlike static or quasi-static models, dynamic models capture time-dependent behaviors, making them essential for replicating fast-reaching motions, as is the case for the elephant trunk.

The main contribution of this work lies in the derivation of a set of stereotypical linear laws via multilinear correlation analysis, which establishes a relationship between the trunk’s shape parameters, segment curvature, and segment length, and the internal forces exerted by the rods. These laws enable the efficient estimation of forces required to achieve desired trunk configurations in planar reaching tasks.

The control framework leverages the derived linear laws to estimate the internal actuation forces required to achieve desired trunk configurations, defined by curvature and length parameters. By applying these computed forces within the dynamic model, the system efficiently generates the target motions. While more complex nonlinear formulations could potentially improve accuracy, the simplicity and speed of the linear approach make it particularly appealing for control applications and low-complexity implementations.

These stereotypical laws also reflect underlying biomechanical principles. The negative correlation between radial and longitudinal forces is consistent with the expected antagonistic behavior of muscular hydrostats, while the positive correlation between radial force and segment length supports the idea that radial contraction contributes to axial elongation. Similarly, the association between curvature and differences in longitudinal force confirms the role of differential activation in driving bending.

Beyond its relevance to biological modeling, the proposed framework presents promising implications for robotics, particularly in the control of continuum modular robots with distributed actuation. A direct application of the stereotypical linear laws would be to establish a mapping between a desired robot configuration, characterized by its shape parameters, and the actuation inputs required to achieve it. The ability to infer actuation forces from global shape parameters may offer advantages in reducing computational cost and simplifying motion planning for continuum robotic platforms. We envision that the framework could be implemented on a modular soft robotic arm with distributed actuation, where each segment features independent shape change. Such a system would ideally be equipped with embedded or external sensing, such as vision-based systems, to provide feedback on segment curvature and length. The measured shape parameters, at each time step, can be input into the derived stereotypical laws to compute the corresponding actuation forces, which are then applied to generate the desired motion.

Building on these findings, future research will explore several extensions. First, expanding the 3D model to incorporate oblique muscles would allow for the simulation of twisting and non-planar motions, bringing the model closer to the full kinematic motion range of a real elephant trunk. Additionally, refining the stereotypical laws by incorporating higher-order or nonlinear terms could enhance their predictive accuracy, albeit at the cost of increased complexity. From a robotics perspective, testing the framework on a physical continuum robotic platform would be a significant step toward validating its applicability in real-world scenarios. By integrating these advancements, we aim to further enhance the model’s predictive power while broadening its utility across biomechanics and soft robotics.

Finally, while the current model assumes homogeneous control principles along the trunk, behavioral evidence suggests that different regions may be governed by distinct neuromuscular strategies.^27^ In particular, the proximal and distal parts of the trunk may serve functionally different roles, potentially leading to region-specific control objectives. Incorporating such distinctions could improve the model’s biological plausibility and predictive accuracy, especially in the distal segments. More broadly, the modeling framework could also be adapted for studying human movement or other biological systems with distributed actuation, such as the tongue.

Overall, this study highlights the value of a structured, low-complexity modeling framework for addressing the challenges posed by hyper-redundant biological systems. By identifying simple force-shape relationships and embedding them into a dynamic simulation environment, the framework represents a preliminary step toward a deeper understanding of hyper-redundant biological structures and their potential to inspire novel robotic solutions.

## Resource availability

### Lead contact

Requests for further information and resources should be directed to and will be fulfilled by the lead contact, Camilla Agabiti (camilla.agabiti@santannapisa.it).

### Materials availability

No new materials were generated in this study.

### Data and code availability

• The dataset used in this study is available at: https://doi.org/10.5281/zenodo.17094445.
• The code used is available at: https://doi.org/10.5281/zenodo.15641895.
• Any additional information required to reanalyze the data reported in this article is available from the lead contact upon request.

## Acknowledgments

This work was supported by the European Union’s Horizon 2020 FET-Open program under Grant 863212 through the PROBOSCIS Project and by the Italian Ministry of Foreign Affairs and International Cooperation, project DESTRO grant n. PGR02061.

## Author contributions

Conceptualization, C.A. (lead), E.D. (supporting), E.S. (supporting), C.L. (supporting), A.M.S. (supporting), and E.F. (supporting); formal analysis, C.A.; methodology, C.A.; data curation, P.D., M.M.; writing – original draft, C.A.; writing – review and editing, E.D., E.S., P.D., M.M., C.L., A.M.S., B.M., and E.F.; funding acquisition, M.M. and E.F.; resources, P.D. and M.M.; supervision, E.F.

## Declaration of interests

The authors declare no competing interests.

## Declaration of generative AI and AI-assisted technologies in the writing process

While preparing this work, the authors used ChatGPT to enhance language and readability. The content was subsequently reviewed and edited by the authors, who take full responsibility for the final version of the submitted article.

## STAR★Methods

### Key resources table

**Table:** 

REAGENT or RESOURCE	SOURCE	IDENTIFIER
**Deposited data**		

Elephant trunk trajectory motion data	Laboratory of Artificial and Natural Evolution (LANE), University of Geneva	N/A
Dataset	https://doi.org/10.5281/zenodo.17094445	N/A

**Software and algorithms**		

MATLAB	https://www.mathworks.com/products/matlab.html	N/A
Code	https://doi.org/10.5281/zenodo.15641895	N/A

### Experimental model and study participants details

Here, we describe the data collection and post-processing of elephant trunk motion data during an experimental campaign conducted by research partners of the European Project PROBOSCIS. We then introduce our methodology for the elephant trunk modeling. A focus is then placed on the equations governing the dynamics of a single rod within the 3D structure. We present the resolution approach, which combines numerical integration and optimization techniques, as well as the physical parameters of the model and the model selection based on those parameters. A section on statistical analysis follows, where we describe the multilinear correlation among the relevant variables of the dynamic model, leading to the derivation of stereotypical linear laws mapping rods forces to desired trunk shapes.

#### Behavioral experiments with elephants, data collection and post-processing

The dynamic model is based on motion data collected during an experimental campaign conducted by PROBOSCIS Consortium partners of the University of Geneva in South Africa (Adventure with Elephants, Bela Bela, South Africa). The experiments involved an adult male African elephant (*Loxodonta africana*) trained to use its trunk to reach and grasp objects of varying shapes and sizes. The primary focus of these trials was to investigate the trunk’s manipulation, and overall motion capabilities. The primary focus of these trials was to investigate the trunk’s manipulation, and overall motion capabilities. To capture trunk movements, a marker-based motion capture system (Qualisys) was used to record the 3D positions of reflective markers placed along the dorsal side of the trunk in ten longitudinal rows, spaced approximately equidistantly along its length.^27^ Due to the extreme tactile sensitivity of elephants, particularly on the ventral side and near the tip, markers could not be placed on the underside of the trunk (see Figure S1 in the Appendix). The dorsal surface was thus the only viable area for marker placement, and this constraint was necessary to ensure the animal acceptance of the experimental protocol.

Each trunk section was approximated as an elliptical cross-section, with all markers belonging to a given section fitted to an ellipse. This approximation was chosen as the simplest and most biomechanically relevant representation of the trunk’s cross-sectional geometry.^27^ Each ellipse was uniquely characterized by eight parameters: its 3D center position, Euler angles (ZYX sequence) defining its orientation, and the lengths of its semi-axes. The center points of the fitted ellipses were used to compute the trunk backbone trajectory. As such, both the ventral surface and the backbone are not directly measured but reconstructed geometrically from dorsal markers. Following data acquisition, the recorded movements were clustered to isolate sequences in which the elephant performed reaching motions. These were defined as movements where the trunk approached a target in space, stopping just before interacting with the object. Four distinct classes of reaching movements were identified: i) reaching through bending, ii) reaching through combination of elongation and bending, iii) reaching through twisting and iv) reaching through combination of twisting and bending. Since our dynamic model focuses on planar reaching motions, only the bending and bending + elongation classes were considered. These classes were determined by assessing two key geometric parameters: length and curvature. We computed the length $L_{\mathrm{seg}}$ as the distance between two subsequent centers of ellipses rows (see 1), where two subsequent ellipses define a segment:

(Equation 1) $$L_{\text{seg}}=\sqrt{{\left(X_{o}^{i+1}-X_{o}^{i}\right)}^{2}+{\left(Y_{o}^{i+1}-Y_{o}^{i}\right)}^{2}+{\left(Z_{o}^{i+1}-Z_{o}^{i}\right)}^{2}}$$

where $\left(X_{o}^{i},Y_{o}^{i},Z_{o}^{i}\right)$ and $\left(X_{o}^{i+1},Y_{o}^{i+1},Z_{o}^{i+1}\right)$ are the coordinates of the centers of two adjacent ellipses. Length was computed for each of the nine segments defined by the ten marker rows and was tracked throughout all recorded motion sequences.

The curvature K of the trunk was computed as the Euclidean norm of its two curvature components, $K_{\alpha }$ and $K_{\beta }$:

(Equation 2) $$K=\sqrt{K_{\alpha }^{2}+K_{\beta }^{2}}$$

where:

(Equation 3) $$K_{\alpha }=\frac{\alpha }{L},\;K_{\beta }=\frac{\beta }{L}$$

Here, $K_{\alpha }$ represents the ratio of the rotation angle α around the normal axis between two consecutive ellipses to the associated segment arclength, while $K_{\beta }$ represents the ratio of the rotation angle β around the binormal axis to the length. Movements were classified as *bending* if the computed curvature exceeded a predefined threshold in at least one segment. Specifically, we set $K_{\text{thresh,}\;\text{bend}}=20\;\text{deg/m}$, which seemed a reasonable value since the length of each segment is approximately 0.2 m. For a movement to be classified as *bending + elongation*, both segment curvature and length had to surpass their respective thresholds in at least one segment. The elongation threshold was defined as $L_{\text{thresh,}\;\text{elong}}=0.02\;\text{m}$. This length threshold values was determined based on the typical segment lengths observed during the trunk’s resting configuration: thus, the length threshold is 10% of segment length. In total, the bending class consists of four motion sequences, whereas the class combining bending and elongation encompasses eight different movements.

### Method details

#### From elephant trunk anatomy to the 3D mechanical model

The 3D model of the elephant trunk was developed by drawing inspiration from the observed muscular arrangement in real elephant trunks.

The elephant trunk is a highly specialized muscular hydrostat, an organ capable of generating movement without bones or a rigid skeleton. Its function relies entirely on the coordinated activity of muscle fibers and connective tissues.^1^^,^^2^ The trunk musculature is organized in a dense three-dimensional array. Fibers are oriented in three main directions: (1) perpendicular to the longitudinal axis, (2) parallel to it, and (3) oblique around it. Transverse and radial fibers form alternating horizontal and vertical layers, or radiate from the center toward the periphery. Radial and transverse muscles primarily reduce trunk diameter, thereby inducing passive elongation. Longitudinal muscles run along the length of the trunk, mainly in peripheral regions: their unilateral contraction, combined with transverse muscle contraction to keep constant diameter, produces bending. Oblique muscles, which enable the trunk to perform non-planar deformations such as torsion, are arranged in V-shaped patterns: the superficial oblique group directed with its open angle toward the trunk tip, and the deep oblique group with its apex pointing toward the tip. Their fiber angle relative to the longitudinal axis (below or above the critical 54°) determines whether contraction results in shortening or elongation, in addition to torsion. In the case of superficial and deep oblique muscles, which show fiber angles of 20°–30°, shortening is the dominant function.^27^ Muscle groups operate antagonistically: for example, contraction of the dorsal longitudinal group, or the superficial and deep oblique groups on the ventral side causes shortening of the trunk, inducing a passive increase in diameter. Connective tissues play a central role in shape control and elastic recoil during antagonistic movements. Transversal intramuscular fibers sorrounds the nasal passages can store elastic energy when trunk diameter is passively increased, allowing elongation with reduced active force. Regional specialization is another key feature of trunk anatomy. Recent high-resolution segmentation studies have shown that the distal trunk, including the tip and nostrils, exhibits denser and more diverse fascicle arrangements, enabling fine manipulations such as grasping small objects or tool use.^29^ This region also hosts numerous mechanoreceptors and tactile vibrissae, which provide rich sensory input and support delicate object exploration and prehension. In contrast, the proximal segments are more robust and primarily support gross positioning and load-bearing functions. Additionally, the distribution of musculature and connective tissue differs between the dorsal and ventral surfaces, with variations in fiber type, density, and mechanical role. These differences may reflect distinct functional demands: for example, the dorsal region may be more involved in stabilization, while the ventral region contributes to lifting and grasping. These anatomical asymmetries suggest that the assumption of full mechanical and functional homogeneity, while helpful for modeling simplification, represents an abstraction of biological reality. Despite this complexity, elephant trunk motion can often be described using low-dimensional control strategies, characterized by traveling waves of curvature, localized stiffening, and segmental elongation.^27^ Such movement primitives can be effectively represented using intrinsic kinematic variables such as curvature, torsion, and strain, which directly reflect the net mechanical output of underlying muscle activity. This compact representation not only captures the biomechanics of movement but also reveals simplified internal coordination strategies that reduce control dimensionality, consistent with motor optimality principles observed in other biological systems.

To replicate these biomechanical features, our 3D dynamic model does not aim to replicate each individual muscle group anatomically, but rather to capture the primary mechanical effects that muscle activations produce on the trunk. The analyzed trunk movements involve macroscopic deformations of stretching and multi-directional planar bending.

The model includes the representation of longitudinal and radial muscles, as well as transverse-oriented connective tissues (see Figure S2 in the Appendix). The trunk is implemented as a structure composed of discrete point masses (nodes), interconnected by axially loaded rods representing simplified muscle-like units. Mass is lumped at the nodes, and the rod arrangement reflects the functional organization of trunk musculature. The structure is divided into nine distinct segments, with endpoints corresponding to the marker rows used in motion capture. Each segment is modeled as a truncated pyramid with non-parallel rectangular bases (see Figure S2), allowing for a structured yet flexible representation of trunk deformations in response to internal forces.

#### Governing equations of a single rod

Before presenting the dynamic equations governing the motion of the entire 3D structure, we introduce the equations describing the dynamics of each individual rod constituting the system. The equation of motion for each rod is derived from Newton’s Second Law (see Equation 4), where each element is defined as a rod connecting two nodes (see Figure S3 in the Appendix).

Solving Equation 4 yields the position of each node over time:

(Equation 4) $$\left[M\right]\ddot{q}+\left[K\right]\left(q\right)=\left[F_{\text{ext}}\right]$$

where [M] is the mass matrix associated with each element. Given the discrete point mass representation, [M] is a 6×6 diagonal matrix, where each mass is repeated three times to account for its decomposition along the X, Y, and Z spatial coordinates. $\ddot{q}$ is the vector of node accelerations (in X, Y, and Z directions) and contains the acceleration components of the two nodes within a single element. q represents the vector of node displacements, $\left[F_{\text{ext}}\right]$ is the external force vector, which, in this model, consists of gravitational forces acting on the system. [K](q) accounts for the elastic properties of the system and can be subdivided into two components: passive and active elasticity.^17^ This term can be rewritten as:

(Equation 5) $$\left[K\right]\left(q\right)={\left[K_{0}\right]}_{\text{loc}}\left(q\right)+\left[F_{\text{act}}\right]$$

Here, ${\left[K_{0}\right]}_{\text{loc}}$ is the local stiffness matrix of each rod, defined in the local reference frame. Since all forces acting on the rods are expressed relative to a global reference frame, the stiffness matrix must also be transformed accordingly, as illustrated in Figure S4 in the Appendix.

The rod elements in this model can only sustain axial loads. However, since each rod connects two nodes acting as hinges, it can transmit forces with components along the X, Y, and Z axes. Consequently, the displacement of each node is described by translations in 3D space. The local stiffness matrix ${\left[K_{0}\right]}_{\text{loc}}$ of a single rod is a 6×6 matrix. The global stiffness matrix is obtained by applying a rotation matrix [R] to transform ${\left[K_{0}\right]}_{\text{loc}}$ into the global reference frame (6):

(Equation 6) $${\left[K_{0}\right]}_{\text{glob}}={\left[R\right]}^{T}{\left[K_{0}\right]}_{\text{loc}}\left[R\right]$$

where the elements of [R] contain the direction cosines that define the orientation of the local reference frame with respect to the global frame (see Figure S4 in the Appendix).

The active force vector $\left[F_{\text{act}}\right]$ contains the internal forces components transmitted along the rods. Since the rods are assumed to sustain only axial loads, these forces are also strictly axial. A schematic representation of the active forces within a generic section i of the structure is shown in Figure S5 (Appendix).

The node at the center of the section is denoted as O, while P,B,Q, and A are the outer nodes. The resultant force acting on each node is derived from the cumulative effect of all rods converging at that node, as described by 7–8:

(Equation 7) $${\vec{F}}_{\text{act,}\;\text{tot}}^{O_{i}}={\vec{F}}_{\text{act}}^{P_{i}O_{i}}+{\vec{F}}_{\text{act}}^{B_{i}O_{i}}+{\vec{F}}_{\text{act}}^{Q_{i}O_{i}}+{\vec{F}}_{\text{act}}^{A_{i}O_{i}}$$

(Equation 8) $${\vec{F}}_{\text{act,}\;\text{tot}}^{P_{i}}={\vec{F}}_{\text{act}}^{O_{i}P_{i}}+{\vec{F}}_{\text{act}}^{P_{i+1}P_{i}}+{\vec{F}}_{\text{act}}^{P_{i-1}P_{i}}$$

Equation 7 describes the resultant force exerted by the radial rods $O_{i}P_{i},O_{i}B_{i},O_{i}Q_{i},O_{i}A_{i}$ on the central node O of a generic section i. Equation 8 describes the forces exerted by the longitudinal rods $P_{i-1}P_{i}$ and $P_{i}P_{i+1}$, along with the radial rod $O_{i}P_{i}$, on node P of section i. The final governing equation for the full system, incorporating all nodes and forces, is expressed as:

(Equation 9) $$\left[M\right]\ddot{q}+{\left[K_{0}\right]}_{\text{glob}}\left(q\right)+F_{\text{act}}=\left[F_{\text{ext}}\right]$$

Equation 9 contains the displacements, accelerations, and forces of all nodes within the system, while the mass and stiffness matrices are represented in their assembled form. To mathematically describe the interconnections among all elements of the structure, it is necessary to establish N equilibrium equations and n consistency equations, where N is the total number of kinematic variables in the structure and n represents the static variables. Given the full structure consists of 50 nodes, the system’s degrees of freedom (DoFs) are represented as.

• $\ddot{q}$ is a 150×1 vector containing the X, Y, and Z acceleration components for all 50 nodes.
• q is a 150×1 vector containing the displacement components in X, Y, and Z.
• $\left[F_{\text{ext}}\right]$ is a 150×1 vector containing the gravitational forces acting on all system nodes.
• [M] is the global mass matrix, which is a 150×150 diagonal matrix under the lumped mass assumption.
• ${\left[K_{0}\right]}_{\text{glob}}$ is the global stiffness matrix, constructed by assembling individual element stiffness matrices. The final global stiffness matrix has a dimension of 150×150.

This assembly process establishes a connection between the element-level equations and the overall structural behavior, enabling the system to properly model the dynamic response of the elephant trunk.

#### Optimization problem

The dynamic equation of motion is solved using numerical integration. We employed MATLAB ODE45 solver, which implements a Runge-Kutta method with a variable time step for efficient computation. Through numerical integration, the acceleration vector $\ddot{q}$ is integrated to obtain velocities $\dot{q}$, and a second integration determines positions q. Since the number of unknowns exceeds the number of independent equations, an additional strategy is required to compute the active force vector $F_{\text{act}}$. To address this, numerical integration is coupled with an optimization framework leveraging experimental trajectory data of elephant trunk motion. The experimental displacement of ellipses points is compared to the displacement of corresponding nodes in simulation, computed as the difference between final and initial positions in the X, Y, and Z coordinates. The optimization problem is formulated to minimize the discrepancy between model-predicted and experimentally observed node displacements:

(Equation 10) $$f_{\text{min}}=\overset{N}{\underset{i=1}{\sum }}{\left\|\Delta q_{\text{model},i}-\Delta q_{\text{real},i}\right\|}^{2}$$

The optimization process determines the optimal active forces $F_{\text{act}}$, which are then applied to replicate trunk movements and predict trajectories. The problem is solved using MATLAB lsqnonlin function from the Optimization Toolbox, designed for nonlinear least-squares problems. The active force vector $F_{\text{act}}$ is computed dynamically at each simulation timestep. The simulation runs at the same sampling frequency as the experimental data, ensuring consistency in the dimension of $F_{\text{act}}$ across simulation steps. At each timestep, $F_{\text{act}}$ is updated as the minimization function is re-evaluated iteratively.

#### Physical parameters of the model

The dynamic model of the elephant trunk is based on physical parameters approximating those of a typical *Loxodonta africana* trunk. With a total length of 185 cm and a mass of approximately 100 kg, the trunk exhibits a non-homogeneous mass distribution, with mass concentrated proximally and decreasing distally. This distribution is approximated by dividing the trunk into ten truncated cone-shaped sub-volumes, where volume and density are used to estimate mass allocation. The total mass is then distributed among fifty discrete nodes, each assigned a mass value reflecting the real trunk’s distribution (see Figure S6 in the Appendix). In each truncated cone section, mass is distributed proportionally to its volume. Since each section contains five nodes, the assigned mass per node is obtained by dividing the section’s total mass by five. The trunk tip (point 10) is excluded from the model due to the absence of motion capture data, as markers could not be placed in that region. Due to limited data on elephant trunk muscle stiffness and elasticity, we adopt an apparent Young’s modulus of $E=1\times 10^{6}$ N/m^2^, following the estimation in.^33^ This value, derived from a continuum static model, is assumed constant across the trunk for simplicity. However, since trunk stiffness varies between longitudinal and radial directions, this approximation introduces some modeling error.

#### Model selection

A sensitivity analysis was conducted to assess the impact of variations in the Young’s modulus of trunk muscles on the 3D dynamic model. This analysis considered the stiffness of longitudinal and radial muscles, as well as connective tissue. Dagenais et al.^27^ observed that elephants use recurrent movement strategies, with a curvature front propagating from the trunk tip and a joint-like motion forming a sharp bend at the midpoint. These patterns suggest a modular trunk behavior, where the proximal region is stiffer for positioning, while the distal region is more flexible for dexterous tasks. Based on this modular approach, we divided the trunk into three sub-volumes, proximal, middle, and distal, each containing three of the nine modeled sub-volumes. The model’s sensitivity to variations in Young’s modulus was then analyzed across these regions, considering both small and significant changes in stiffness. Table S1 in the Appendix summarizes the tested values.

Set 1 represents a scenario with minor variations in Young’s modulus compared to the initially assumed value, while Sets 2, 3, and 4 introduce progressively larger variations. This approach allows us to assess the model’s response to different stiffness distributions and identify their impact on trunk dynamics.

### Quantification and statistical analysis

#### Multilinear correlation analysis of active forces and trunk shape parameters

The active forces exerted by the rods in the 3D trunk model are key parameters influencing trunk dynamics. These forces act on the nodes where rods converge, inducing displacements and shaping trunk motion. We analyzed active force behavior in two deformation classes: bending and bending + elongation. Following the muscle synergies described in Dagenais et al.,^27^ bending results from differential activation of longitudinal muscles, while elongation is primarily driven by radial muscle contraction. To explore these relationships, we identified five key variables for each trunk segment.

• Curvature (K) – segment bending angle per unit length
• Force difference $\left(\Delta F_{L}\right)$ – between dorsal and ventral longitudinal rods
• Segment length (L)
• Radial rod force $\left(F_{R}\right)$
• Mean longitudinal force $\left(F_{m\text{\_}L}\right)$ – average force of dorsal and ventral longitudinal rods

A multilinear correlation analysis was performed to investigate the relationships between trunk shape parameters (K and L) and internal forces ($\Delta F_{L}$, $F_{R}$, and $F_{m\text{\_}L}$). These relationships were modeled using the general multilinear equation:

(Equation 11) $$Y=\beta_{0}+\beta_{1}X_{1}+\beta_{2}X_{2}+\beta_{3}X_{3}+\epsilon$$

where Y is the dependent variable, $X_{1},X_{2},X_{3}$ are the predictors, $\beta_{0}$ is the intercept coefficient, $\beta_{1},\beta_{2},\beta_{3}$ are regression coefficients, and ϵ is the residual error. The model accuracy was assessed using the R-squared $\left(R^{2}\right)$ metric, indicating the variance explained by predictors, and the Root-Mean-Square Error (RMSE), measuring the predictive accuracy. We defined two separate laws, either for the segment curvature or its length, in order to find their dependencies on the internal force parameters. We formalized the law for the curvature and length of the i-th segment as expressed in Equations 12 and 13:

(Equation 12) $$K^{i}=\beta_{0}^{K}+\beta_{1}^{K}\Delta F_{L}^{i}+\beta_{2}^{K}F_{R}^{i}+\beta_{3}^{K}F_{m\text{\_}L}^{i}+\epsilon_{K}^{i}$$

(Equation 13) $$L^{i}=\beta_{0}^{L}+\beta_{1}^{L}\Delta F_{L}^{i}+\beta_{2}^{L}F_{R}^{i}+\beta_{3}^{L}F_{m\text{\_}L}^{i}+\epsilon_{L}^{i}$$

Additionally, a relationship among internal forces was obtained (Equation 14):

(Equation 14) $$F_{\text{rad}}^{i}=\beta_{0}^{F}+\beta_{1}^{F}\Delta F_{L}^{i}+\beta_{3}^{F}F_{m\text{\_}L}^{i}+\epsilon_{F}^{i}$$

This determined system allows for computing internal rod forces.

#### Mapping trunk shape evolution to force coordination in planar reaching

We analyzed the relationship between muscle-like rod forces and trunk shape parameters using the estimated multilinear laws. The input data consisted of the initial and final configurations of each trunk segment, defined by curvature $\left(K_{\text{in}}^{i},K_{\text{fin}}^{i}\right)$ and length $\left(L_{\text{in}}^{i},L_{\text{fin}}^{i}\right)$, along with the trial duration T. To model the transition between these configurations, we interpolated curvature and length at a sampling frequency $f_{s}=100$ Hz using a convex combination approach. The curvature and length of segment i at time $t_{j}$ were computed as in Equations 15 and 16:

(Equation 15) $$K_{t_{j}}^{i}=K_{\text{in}}^{i}\frac{T-t_{j}}{T}+K_{\text{fin}}^{i}\frac{t_{j}}{T}$$

(Equation 16) $$L_{t_{j}}^{i}=L_{\text{in}}^{i}\frac{T-t_{j}}{T}+L_{\text{fin}}^{i}\frac{t_{j}}{T}$$

where $t_{j}=j/f_{s}$ for $0\leq j<Tf_{s}$. This method generates intermediate segment curvature and length values between the initial and final trunk configurations. At each time step $t_{j}$, the multilinear correlation laws (Equations 12, 13, and 14) were used to compute unknown internal rod forces for each trunk segment. These computed forces were then applied as constant inputs to the forward dynamic model. During each time interval $t_{j}\leq t<t_{j+1}$, the dynamic model used these inputs to simulate the trunk’s planar reaching movements.
