# Supplementary material for: Stereotypical force patterns of the elephant trunk in planar reaching movements
Source: iScience. 2026 Feb 23;29(4):115108. doi: 10.1016/j.isci.2026.115108 (PMC13010104; doi:10.1016/j.isci.2026.115108)
Supplement: Document S1. Figures S1–S11 and Tables S1–S6 [file mmc1.pdf]

iScience, Volume *29*

Supplemental information

Stereotypical force patterns of the elephant trunk in planar reaching movements

Camilla Agabiti, Enrico Donato, Elisa Setti, Paule Dagenais, Michel C. Milinkovitch, Cecilia Laschi, Angelo Maria Sabatini, Barbara Mazzolai, and Egidio Falotico

Supplemental Figures and Tables

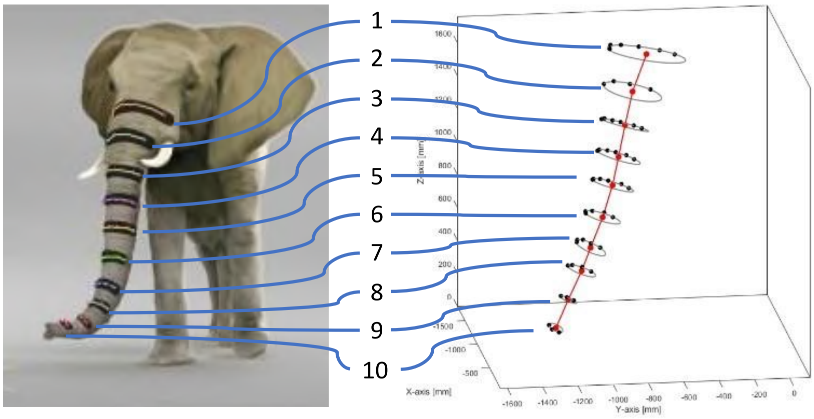

Figure S1. *Left: schematic representation of marker rows distributed along the elephant trunk (adapted from Dagenais et al., (2021). Right: virtual reconstruction of the trunk based on ellipses fitted to each marker row*

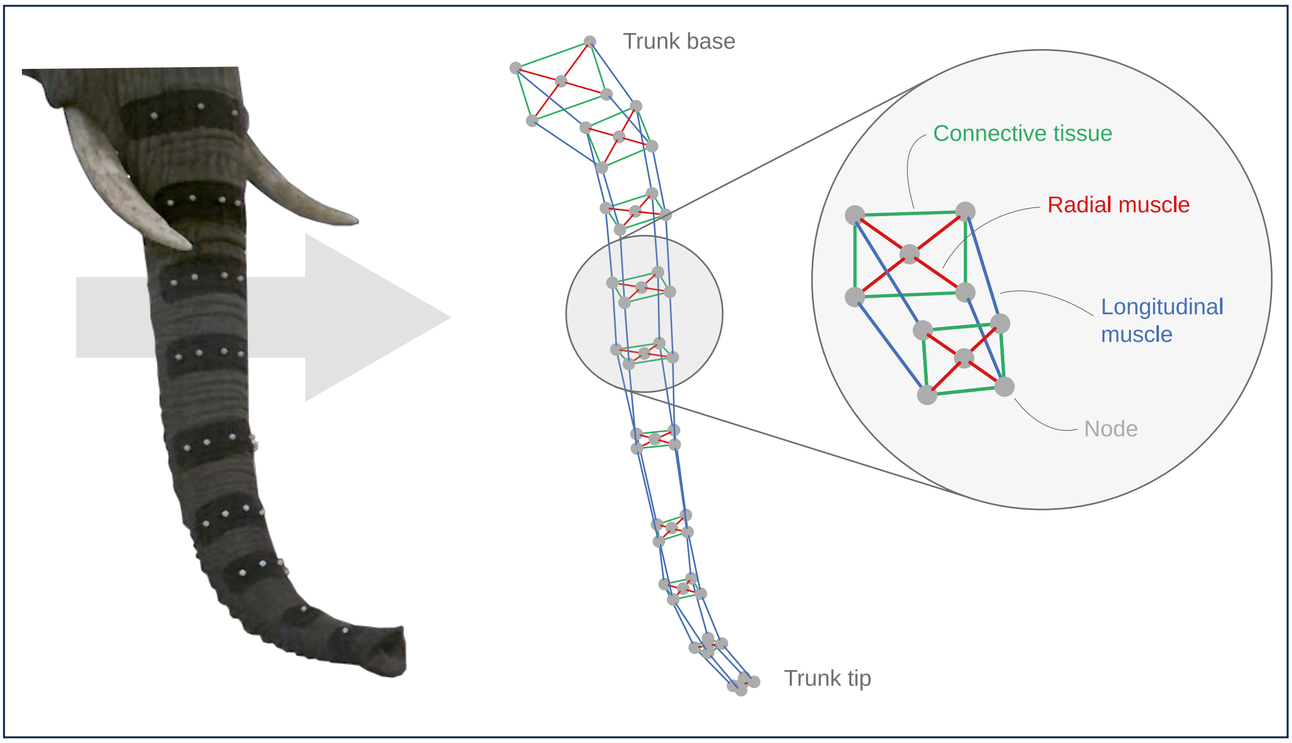

Figure S2. *From the left: elephant trunk with markers rows. Centre: elephant trunk modeled through interconnected rods and nodes*

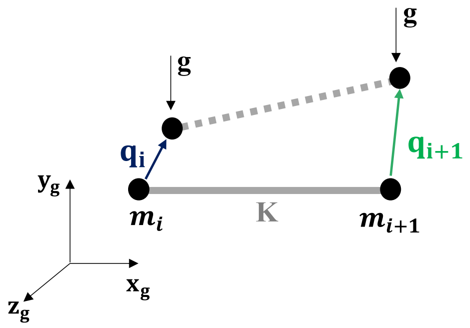

Figure S3. *Schematic of 1D rod element with two hinges at its endpoints. K is the stiffness, m the mass, g the gravitational force, and q the node displacement. Rod element shown in the global reference frame*

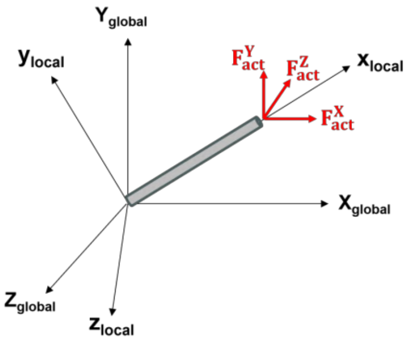

Figure S4. *Global and local reference frames in a rod element*

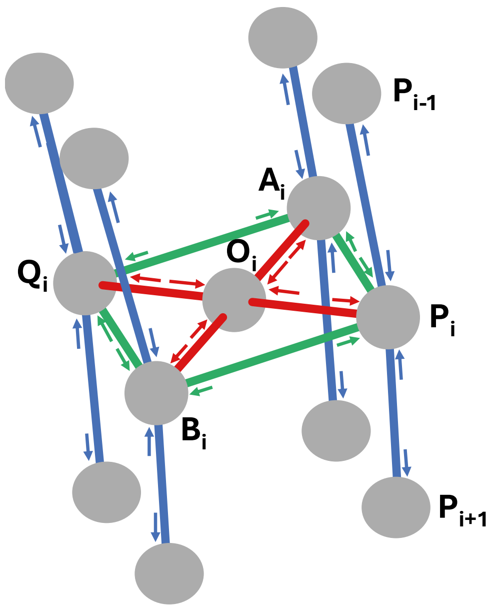

Figure S5. *Schematic representation of active forces (arrows) on a generic trunk section i acting on nodes O, P, B, Q, A*

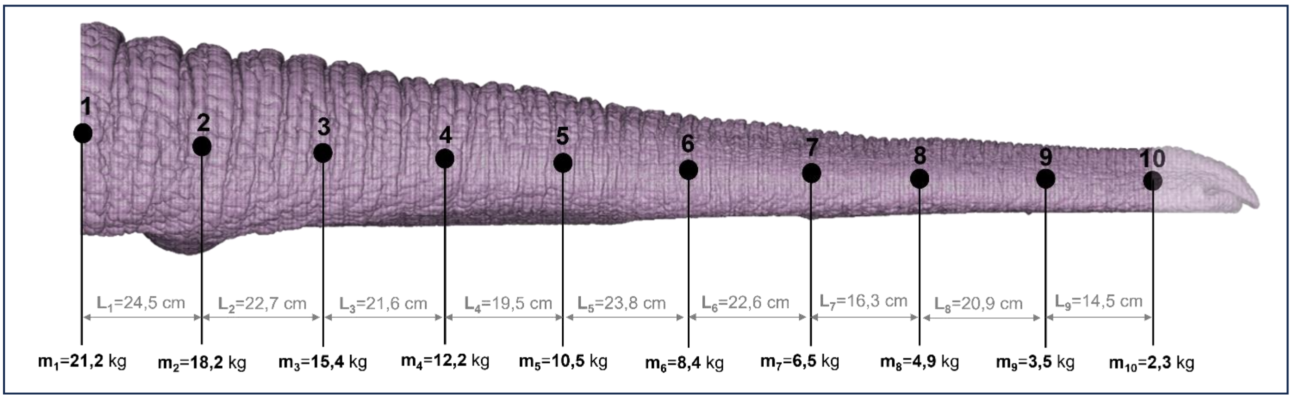

Figure S6. *Sketch showing the mass distribution at trunk sections and lengths of trunk segments*

Set	Proximal segments	Middle segments	Distal segments
1	$E_{prox} = 1e6\ N/m^2$	$E_{mid} = 5e5\ N/m^2$	$E_{dist} = 1e5\ N/m^2$
2	$E_{prox} = 1e6\ N/m^2$	$E_{mid} = 1e5\ N/m^2$	$E_{dist} = 1e4\ N/m^2$
3	$E_{prox} = 1e7\ N/m^2$	$E_{mid} = 1e6\ N/m^2$	$E_{dist} = 1e5\ N/m^2$
4	$E_{prox} = 5e7\ N/m^2$	$E_{mid} = 5e6\ N/m^2$	$E_{dist} = 1e6\ N/m^2$

Table S1. *Young's modulus values used in the sensitivity analysis.*

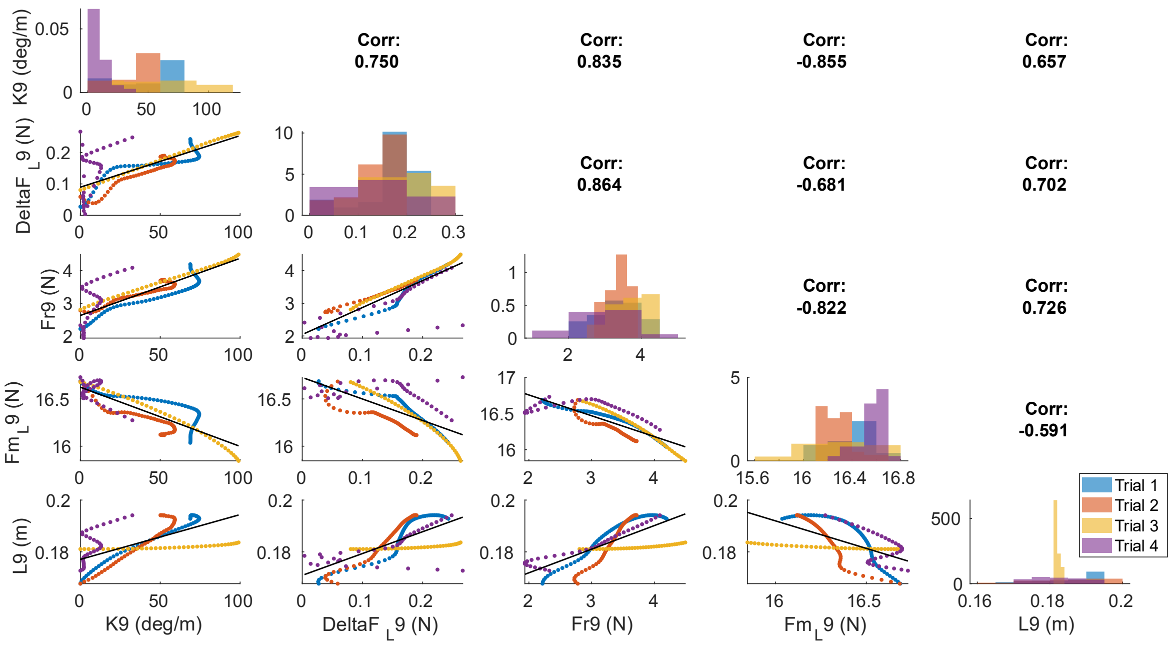

***Figure S7*. Pairwise linear correlation among the variables in the analysis for trunk segment #4 in bending (B) movements.** *The considered variables are: segment curvature (K), segment length (L), difference between longitudinal dorsal and ventral rod forces (ΔF_L), mean force between longitudinal dorsal and ventral rod forces (F_{m_L}), and radial rod force (F_R). The diagonal histograms display the distribution of each variable across all four B trials. The scatterplots illustrate the relationships between each pair of variables, with overlaid linear regression lines and color-coded trajectories for the individual trials.*

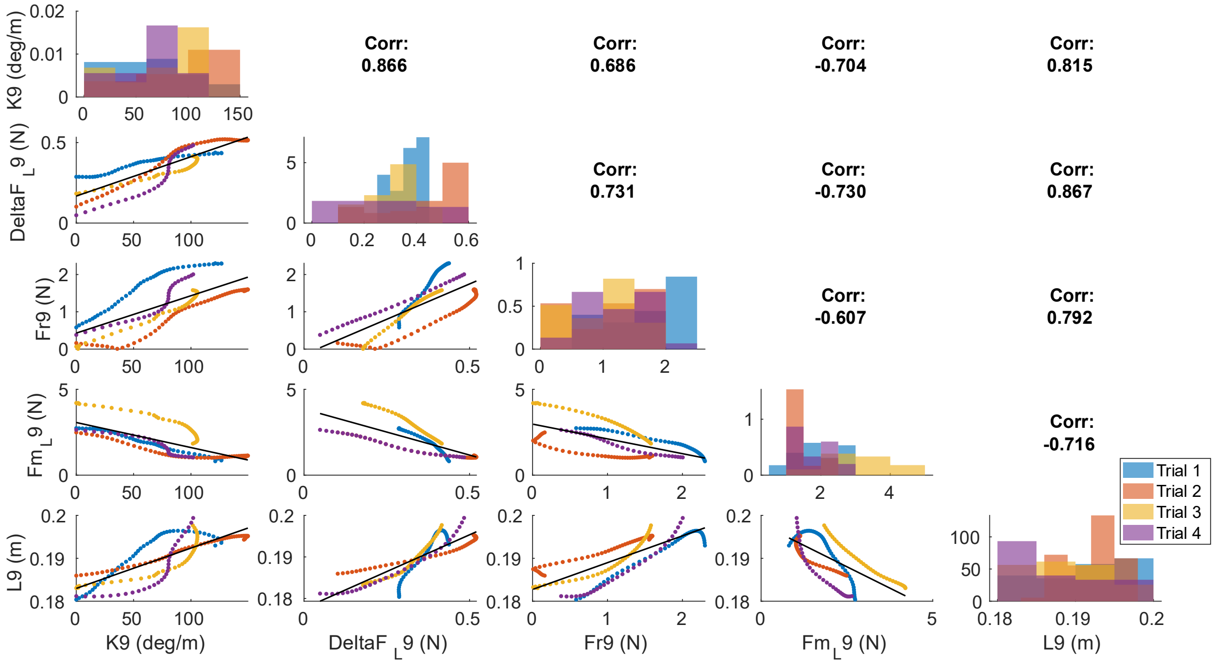

Figure S8. Pairwise linear correlation among the variables in the analysis for trunk segment #9 in bending (B) movements. *The considered variables are: segment curvature (K), segment length (L), difference between longitudinal dorsal and ventral rod forces (ΔF_L), mean force between longitudinal dorsal and ventral rod forces (F_{m_L}), and radial rod force (F_R). Diagonal histograms and scatterplots are shown as in Figure S1*

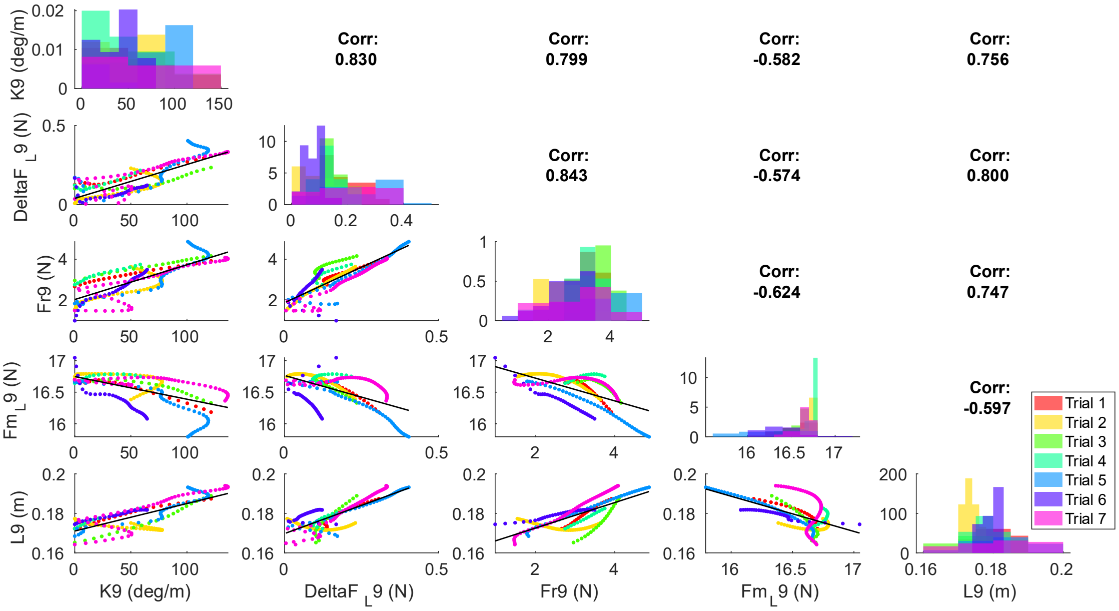

***Figure S9**. **Pairwise linear correlation among the variables in the analysis for trunk segment #4 in combined bending /elongation (BE) movements.** The considered variables are: segment curvature (K), segment length (L), difference between longitudinal dorsal and ventral rod forces (ΔF_L), mean force between longitudinal dorsal and ventral rod forces (F_{m_L}), and radial rod force (F_R). Diagonal histograms and scatterplots are shown as in Figure S1*

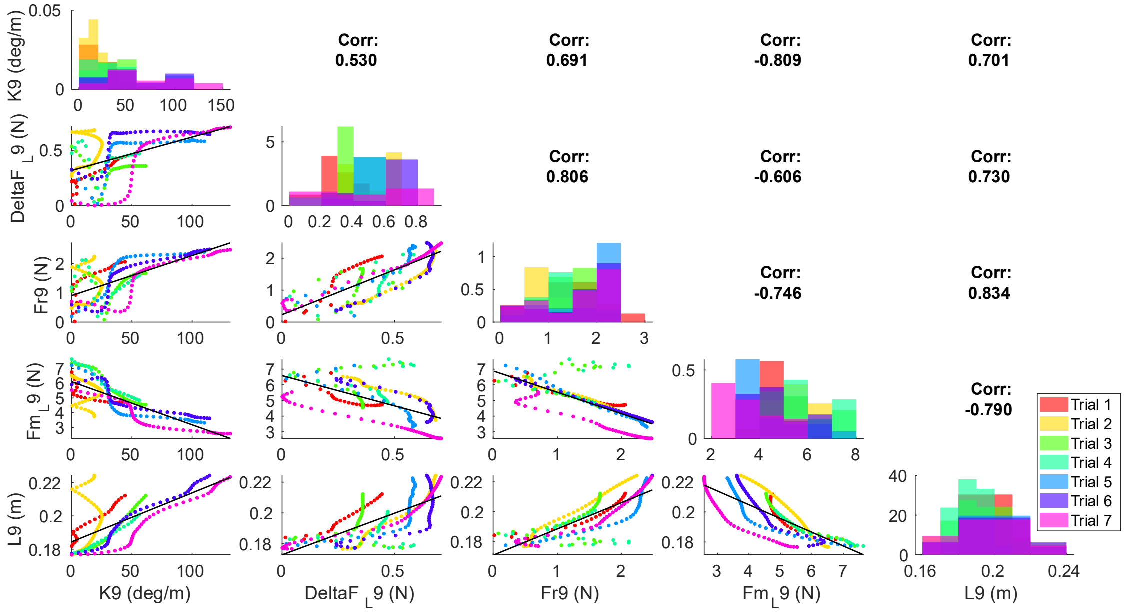

***Figure S10**. **Pairwise linear correlation among the variables in the analysis for trunk segment #9 in combined bending/elongation (BE) movements.** The considered variables are: segment curvature (K), segment length (L), difference between longitudinal dorsal and ventral rod forces (ΔF_L), mean force between longitudinal dorsal and ventral rod forces (F_{m_L}), and radial rod force (F_R). Diagonal histograms and scatterplots are shown as in Figure S1*

Segment	Bending (B)	Bending + Elongation (BE)
1	$K_1 = 11.6 + 368\Delta F_{L1} + 2.20F_{R1} - 2.01F_{mL1}$	$K_1 = 97.6 + 208\Delta F_{L1} + 2.52F_{R1} - 6.53F_{mL1}$
2	$K_2 = 5.42 + 375.6\Delta F_{L2} + 1.51F_{R2} - 1.529F_{mL2}$	$K_2 = 81.1 + 236\Delta F_{L2} + 2.32F_{R2} - 5.61F_{mL2}$
3	$K_3 = 15.3 + 369\Delta F_{L3} + 1.80F_{R3} - 2.21F_{mL3}$	$K_3 = 87.7 + 235\Delta F_{L3} + 2.64 - 6.121F_{mL3}$
4	$K_4 = 17.5 + 373\Delta F_{L4} + 1.51F_{R4} - 2.34F_{mL4}$	$K_4 = 85.7 + 211\Delta F_{L4} + 2.31F_{R4} - 5.82F_{mL4}$
5	$K_5 = 10.9 + 362\Delta F_{L5} + 2.55F_{R5} - 2.02F_{mL5}$	$K_5 = 72.2 + 216\Delta F_{L5} + 2.10F_{R5} - 5.20F_{mL5}$
6	$K_6 = 3.50 + 257\Delta F_{L6} + 1.53F_{R6} - 6.01F_{mL6}$	$K_6 = 36.7 + 157\Delta F_{L6} + 2.11F_{R6} - 5.44F_{mL6}$
7	$K_7 = 0.50 + 258\Delta F_{L7} + 2.41F_{R7} - 5.50F_{mL7}$	$K_7 = 39.5 + 231\Delta F_{L7} + 2.25F_{R7} - 5.81F_{mL7}$
8	$K_8 = -1.3 + 262\Delta F_{L8} + 2.20F_{R8} - 5.21F_{mL8}$	$K_8 = 40.5 + 150\Delta F_{L8} + 3.10F_{R8} - 6.11F_{mL8}$
9	$K_9 = -3.70 + 264\Delta F_{L9} + 2.60F_{R9} - 4.82F_{mL9}$	$K_9 = 41.7 + 147\Delta F_{L9} + 3.40F_{R9} - 6.21F_{mL9}$

***Table S2. Stereotypical laws obtained from the multilinear correlation for segment curvature K**. Laws relating segment curvature to the internal rod forces (difference between longitudinal dorsal and ventral rod forces (ΔF_L), mean force between longitudinal dorsal and ventral rod forces (F_{m_L}), and radial rod force (F_R)). Laws reported for bending (B) and bending/elongation (BE) movements, for each trunk segment*

Segment	Bending (B)	Bending + Elongation (BE)
1	$L_1 = 0.46 + 0.0063\Delta F_{L1} + 0.011F_{R1} - 0.0050F_{mL1}$	$L_1 = 0.150 + 0.0011\Delta F_{L1} + 0.00069F_{R1} - 0.0179F_{mL1}$
2	$L_2 = 0.23 + 0.021\Delta F_{L2} + 0.022F_{R2} - 0.001F_{mL2}$	$L_2 = 0.145 + 0.00090\Delta F_{L2} + 0.00506F_{R2} - 0.00627F_{mL2}$
3	$L_3 = -0.060 + 0.0015\Delta F_{L3} + 0.011F_{R3} - 0.013F_{mL3}$	$L_3 = 0.135 + 0.0208\Delta F_{L3} + 0.0032F_{R3} - 0.0060F_{mL3}$
4	$L_4 = 0.14 + 0.27\Delta F_{L4} + 0.0031F_{R4} - 0.019F_{mL4}$	$L_4 = 0.145 + 0.011\Delta F_{L4} + 0.0025F_{R4} - 0.0104F_{mL4}$
5	$L_5 = 0.16 + 0.015\Delta F_{L5} + 0.014F_{R5} - 0.0038F_{mL5}$	$L_5 = 0.147 + 0.0042\Delta F_{L5} + 0.0027F_{R5} - 0.0117F_{mL5}$
6	$L_6 = 0.42 + 0.20\Delta F_{L6} + 0.21F_{R6} - 0.021F_{mL6}$	$L_6 = 0.145 + 0.0070\Delta F_{L6} + 0.0042F_{R6} - 0.0068F_{mL6}$
7	$L_7 = 0.21 + 0.0031\Delta F_{L7} + 0.0072F_{R7} - 0.0043F_{mL7}$	$L_7 = 0.173 + 0.0239\Delta F_{L7} + 0.0043F_{R7} - 0.0054F_{mL7}$
8	$L_8 = -0.18 + 0.19\Delta F_{L8} + 0.065F_{R8} - 0.13F_{mL8}$	$L_8 = 0.180 + 0.0321\Delta F_{L8} + 0.020F_{R8} - 0.0056F_{mL8}$
9	$L_9 = 0.36 + 0.045\Delta F_{L9} + 0.027F_{R9} - 0.068F_{mL9}$	$L_9 = 0.180 + 0.0395\Delta F_{L9} + 0.0024F_{R9} - 0.0049F_{mL9}$

***Table S3. Stereotypical laws for segment length (L).** Laws obtained from the multilinear correlation, relating segment length (L) to the internal rod forces (difference between longitudinal dorsal and ventral rod forces (ΔF_L), mean force between longitudinal dorsal and ventral rod forces (F_{m_L}), and radial rod force (F_R)). Laws reported for bending (B) and bending/elongation (BE) movements, for each trunk segment.*

Segment	Bending (B)	Bending + Elongation (BE)
1	$F_{R1} = 14.9 + 7.0\Delta F_{L1} - 0.81F_{mL1}$	$F_{R1} = 8.62 + 8.75\Delta F_{L1} - 0.52F_{mL1}$
2	$F_{R2} = 16.7 + 6.01\Delta F_{L2} - 0.9F_{mL2}$	$F_{R2} = 10.4 + 8.61\Delta F_{L2} - 1.69F_{mL2}$
3	$F_{R3} = 20.3 + 4.0\Delta F_{L3} - 1.1F_{mL3}$	$F_{R3} = 12.7 + 9.25\Delta F_{L3} - 0.71F_{mL3}$
4	$F_{R4} = 16.7 + 6.11\Delta F_{L4} - 0.9F_{mL4}$	$F_{R4} = 10.2 + 7.92\Delta F_{L4} - 1.63F_{mL4}$
5	$F_{R5} = 22.1 + 3.0\Delta F_{L5} - 1.2F_{mL5}$	$F_{R5} = 8.62 + 9.75\Delta F_{L5} - 0.55F_{mL5}$
6	$F_{R6} = 25.7 + 1.1\Delta F_{L6} - 1.4F_{mL6}$	$F_{R6} = 2.46 + 1.33\Delta F_{L6} - 0.36F_{mL6}$
7	$F_{R7} = 2.4 + 1.0\Delta F_{L7} - 0.4F_{mL7}$	$F_{R7} = 3.23 + 0.67\Delta F_{L7} - 0.45F_{mL7}$
8	$F_{R8} = 1.8 + 2.0\Delta F_{L8} - 0.3F_{mL8}$	$F_{R8} = 2.85 + 1.52\Delta F_{L8} - 0.35F_{mL8}$
9	$F_{R9} = 2.4 + 1.1\Delta F_{L9} - 0.468F_{mL9}$	$F_{R9} = 1.58 + 0.88\Delta F_{L9} - 0.28F_{mL9}$

***Table S4. Stereotypical laws for radial force (F_R).** Laws obtained from the multilinear correlation, relating radial force (F_R) to difference between longitudinal dorsal and ventral rod forces (ΔF_L) and mean force between longitudinal dorsal and ventral rod forces (F_{m_L}). Laws reported for bending (B) and bending/elongation (BE) movements, for each trunk segment.*

	MAE_2D in Bending (B) trials			RMSE in Bending (B) trials		
	**Min [m]**	**Max [m]**	**Median [m]**	**Min [m]**	**Max [m]**	**Median [m]**
**Node 2**	0.0063	0.0121	0.0099	0.0082	0.0166	0.0124
**Node 3**	0.0061	0.0130	0.0106	0.0077	0.0185	0.0137
**Node 4**	0.0102	0.0185	0.0166	0.0137	0.0261	0.0209
**Node 5**	0.0113	0.0243	0.0217	0.0155	0.0344	0.0266
**Node 6**	0.0136	0.0314	0.0275	0.0180	0.0431	0.0335
**Node 7**	0.0148	0.0395	0.0329	0.0186	0.0491	0.0413
**Node 8**	0.0121	0.0493	0.0308	0.0134	0.0602	0.0377
**Node 9**	0.0178	0.0463	0.0236	0.0246	0.0546	0.0286
**Node 10**	0.0224	0.0573	0.0425	0.0318	0.0662	0.0492

Table S5. *Summary of Euclidean 2D Mean Absolute Error (MAE_2D) and Root Mean Squared Error (RMSE) for each node of the trunk during Bending (B) trials. Values include minimum, maximum, and median across all repetitions. A progressive increase in both MAE and RMSE can be observed from proximal (Node 2) to distal (Node 10) regions of the trunk.*

	MAE_2D in Bending-Elongation (BE) trials			RMSE in Bending-Elongation (BE) trials		
	**Min [m]**	**Max [m]**	**Median [m]**	**Min [m]**	**Max [m]**	**Median [m]**
**Node 2**	0.0028	0.0289	0.0059	0.0032	0.0360	0.0084
**Node 3**	0.0038	0.0189	0.0086	0.0049	0.0245	0.0112
**Node 4**	0.0029	0.0187	0.0125	0.0033	0.0240	0.0159
**Node 5**	0.0015	0.0228	0.0176	0.0017	0.0293	0.0236
**Node 6**	0.0084	0.0338	0.0296	0.0124	0.0427	0.0385
**Node 7**	0.0170	0.0451	0.0415	0.0253	0.0549	0.0526
**Node 8**	0.0247	0.0646	0.0430	0.0350	0.0888	0.0577
**Node 9**	0.0323	0.0608	0.0427	0.0366	0.0833	0.0522
**Node 10**	0.0309	0.0890	0.0482	0.0434	0.1015	0.0450

Table S6. *Summary of Euclidean 2D Mean Absolute Error (MAE_2D) and Root Mean Squared Error (RMSE) for each node of the trunk during Bending-Elongation (BE) trials. Values include minimum, maximum, and median across all repetitions. Similarly to the B case, a progressive increase in both MAE and RMSE can be observed from proximal to distal nodes.*

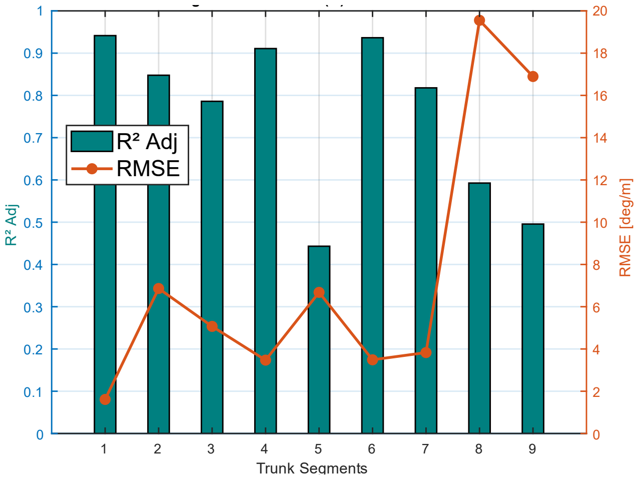

Figure S11. *Adjusted R^2 values and RMSE for the curvature regression model across backbone segments.*
